# Cyclic Deformation Induced Residual Stress Evolution and 3D Short Fatigue Crack Growth Investigated by Advanced Synchrotron Tomography Techniques

**DOI:** 10.3390/ma14061562

**Published:** 2021-03-22

**Authors:** Benjamin Dönges, Melanie Syha, Anne K. Hüsecken, Ullrich Pietsch, Wolfgang Ludwig, Ulrich Krupp, Hans-Jürgen Christ

**Affiliations:** 1 Institut für Werkstofftechnik, Universität Siegen, D-57068 Siegen, Germany; benjamin.doenges@gmx.de; 2 Institut für Angewandte Materialien, Karlsruher Institut für Technologie, D-76131 Karlsruhe, Germany; melanie.syha@ds-ing.net; 3 European Synchrotron Radiation Facility, F-38043 Grenoble, France; wolfgang.ludwig@esrf.fr; 4 Festkörperphysik, Department Physik, Universität Siegen, D-57068 Siegen, Germany; anne.huesecken@gmx.de (A.K.H.); pietsch@physik.uni-siegen.de (U.P.); 5 Institut für Eisenhüttenkunde, RWTH Aachen, D-52072 Aachen, Germany; krupp@iehk.rwth-aachen.de

**Keywords:** synchrotron tomography, short fatigue crack growth, residual stress, crystal plasticity, very high cycle fatigue

## Abstract

Diffraction and phase contrast tomography techniques were successfully applied to an austenitic–ferritic duplex stainless steel representing exemplarily a metallic material containing two phases with different crystal structures. The reconstructed volumes of both phases were discretized by finite elements. A crystal plasticity finite-element analysis was executed in order to simulate the development of the experimentally determined first and second order residual stresses, which built up due to the manufacturing process of the material. Cyclic deformation simulations showed the single-grain-resolved evolution of initial residual stresses in both phases and were found to be in good agreement with the experimental results. Solely in ferritic grains, residual stresses built up due to cyclic deformation, which promoted crack nucleation in this phase. Furthermore, phase contrast tomography was applied in order to analyze the mechanisms of fatigue crack nucleation and short fatigue crack propagation three-dimensionally and nondestructively. The results clearly showed the significance of microstructural barriers for short fatigue crack growth at the surface, as well as into the material. The investigation presented aims for a better understanding of the three-dimensional mechanisms governing short fatigue crack propagation and, in particular, the effect of residual stresses on these mechanisms. The final goal was to generate tailored microstructures for improved fatigue resistance and enhanced fatigue life.

## 1. Introduction

The failure of safety-relevant components by fatigue of materials in form of crack initiation and propagation caused by periodic loading far below the static strength of a material may cause unexpected damage events and, thereby, is a thread not just for components and engineering structures but also for human life. It is known that the phases of fatigue crack initiation and propagation can cover more than 90% of the total fatigue life of a material at loading situations close to the conventional fatigue limit [1], and therefore, this fatigue stage can be considered as determining time to failure of a component. Recent investigations have shown the significance of microstructural aspects, such as (i) anisotropic elasticity of grains, (ii) three-dimensional geometry of grain and phase boundaries, and (iii) microscopic residual stresses, for fatigue crack initiation and propagation [2,3].

Three-dimensional characterization of dual phase materials has already been realized by means of serial sectioning electron back scatter diffraction (EBSD) experiments [4,5,6]. However, being a destructive technique, serial sectioning does not provide the possibility to follow the microstructure evolution quasi in situ by means of interrupted fatigue experiments. The combined use of X-ray diffraction contrast tomography and multi-distance phase contrast tomography [7] allows for the unique possibility to reconstruct the three-dimensional microstructure of a two-phase material while simultaneously determining the crystallographic orientation of each grain. Due to the very high resolution achieved in holotomography experiments, a high certainty of the location of inter-phase grain boundaries is achieved. The non-destructive nature of these techniques allows for cyclic deformation with intermittent hold times or slow cycles for grain orientation and grain shape determination.

In a previous study [8], the cyclic deformation-induced change of manufacturing-caused residual stress in the investigated austenitic–ferritic duplex stainless steel was experimentally characterized by means of high energy synchrotron radiation for up to one hundred million load cycles. Due to a higher thermal expansion coefficient of the austenite phase in comparison to the ferrite phase, tensile residual stress is generated in austenite grains and compressive residual stress is generated in ferrite grains as a result of prior quenching from 1050 °C to room temperature as the final part of the manufacturing process of the material. The change of residual stress was investigated by measuring the lattice spacing *d* by means of synchrotron radiation diffraction experiments after predefined numbers of loading cycles (intermittent fatigue test) and determining the relative change of the lattice spacing Δ*d* with respect to the initial lattice spacing before cyclic loading *d_N = 0_*.

**Figure 1:**
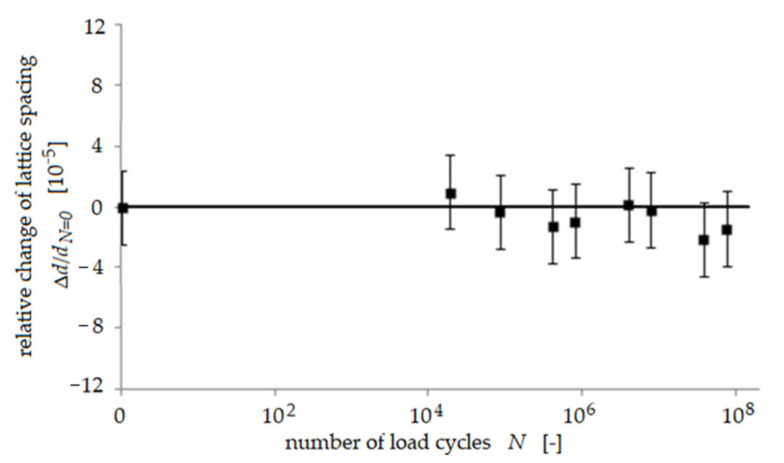
Relative change of lattice spacing *d* in a single grain during cyclic loading at a stress am-plitude of 380 MPa, using the example of an austenite [040] reflection according to [8].

**Figure 2:**
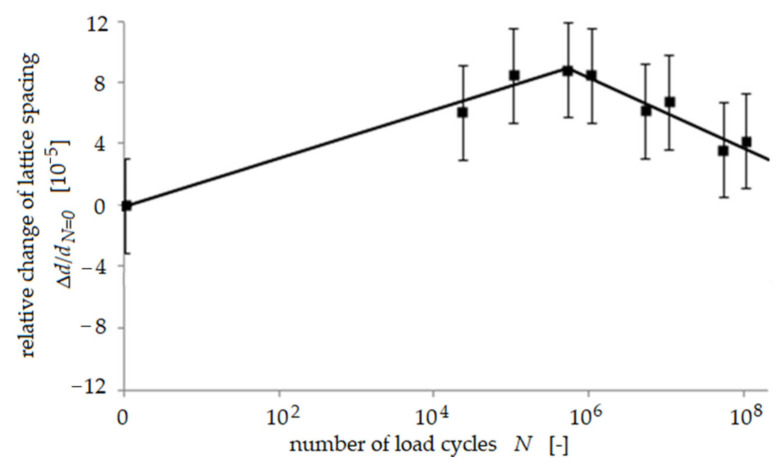
Relative change of lattice spacing *d* in a single grain during cyclic loading at a stress am-plitude of 380 MPa, using the example of a ferrite [031] reflection according to [8].

**Figure 3:**
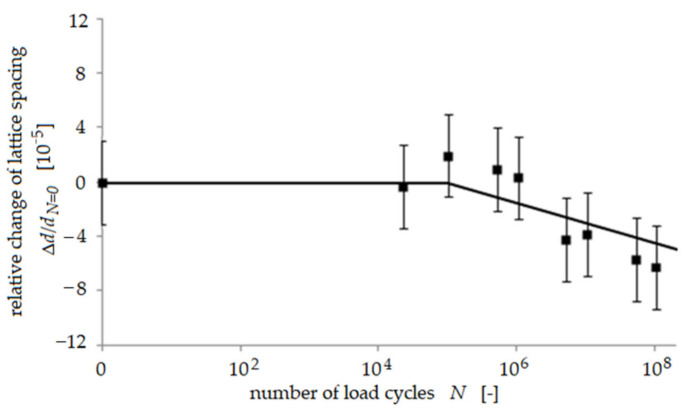
Relative change of lattice spacing *d* in a single grain during cyclic loading at a stress am-plitude of 380 MPa, using the example of another ferrite [031] reflection according to [8].

Only small changes of the relative lattice spacing as a function of the number of load cycles *N* were found in most of the austenite grains and also in some of the ferrite grains (shown exemplarily for one grain in Figure 1), indicating that no change of residual stress occurred in these grains. In about half of all ferrite grains, the lattice spacing was found to increase continuously during the first 5·10^5^ load cycles and, subsequently, to decrease again slightly (Figure 2). Hence, initial compressive residual stress was reduced in these ferrite grains during cyclic deformation. This is a comprehensible effect since it is well-known that plastic deformation usually causes a reduction of initial residual stress. This increase of lattice spacing was not observed in any austenite grain. A third trend showed that the lattice spacing in a few austenite grains appeared to first stay unchanged until 10^5^ load cycles, but then, during subsequent load cycles, the lattice spacing in these grains continuously became smaller (Figure 3), indicating that initial tensile residual stress was reduced in these austenite grains during cyclic deformation, being a comprehensible effect as already stated above. The most interesting observation in this study was that an increase of lattice spacing was found for many ferrite grains, indicating a reduction of the initial compressive residual stress This effect of partial annihilation of compressive residual stress in some ferrite grains is remarkable and seems to promote the observed predominant fatigue crack initiation process in the ferrite phase as described in detail in [9,10]. It should be noted that the saturation or slight reduction of initial tensile residual stress in the austenite grains is in accordance with the observed true fatigue limit found for the material investigated.

The present study shows experimental techniques that enable the nondestructive characterization of three-dimensional polycrystalline microstructures in order to obtain information about the shape and crystallographic orientation of the single grains. Moreover, these techniques enable the investigation of the three-dimensional interaction between short fatigue cracks and grain and phase boundaries. It is shown how this experimental data can be used in the framework of three-dimensional crystal plasticity finite-element simulations to calculate realistically the development of residual stress as a function of the number of load cycles. Such simulations may serve as a basis for a realistic fatigue life assessment model, which may enable the development of tailored microstructures with improved fatigue resistance and enhanced fatigue life in the future.

## 2. Experimental Details

**Figure 4:**
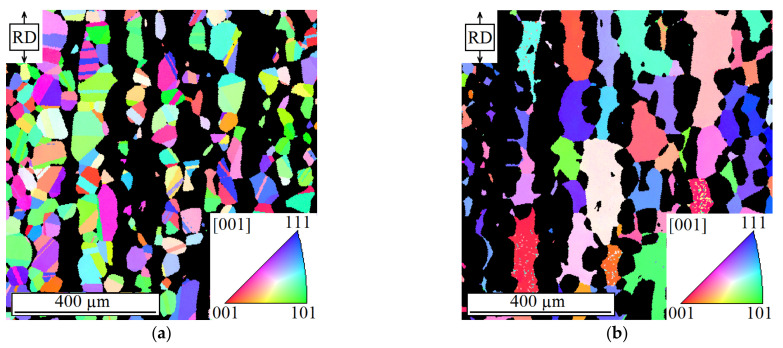
Inverse pole figure maps of (**a**) the austenite phase and (**b**) the ferrite phase of the investigated duplex stainless steel, taken parallel to the rolling direction (vertical) according to [3].

Duplex stainless steels have a high corrosion resistance and relatively high strength, which makes them very attractive for use, e.g., in offshore systems or systems for the chemical and petrochemical industry. The investigated austenitic-ferritic duplex stainless steel 318LN (German designation X2CrNiMoN22-5-3) was delivered as hot rolled and solution annealed bars with a diameter of 25 mm. The material consisted of a fine lamellar microstructure with about 50 vol% austenite and 50 vol% ferrite. The chemical composition of the material is shown in Table 1. In order to ease the experimental investigations, a grain coarsening was carried out by annealing the material at 1250 °C for 4 h followed by a cooling down to 1050 °C within 3 h at constant cooling rate. Subsequently, the material was quenched in water. By means of this heat treatment, the initial volume fraction of both phases was maintained, and the mean grain diameter of the austenite phase and the ferrite phase was increased to 33 µm and 46 µm, respectively. Figure 4 shows the microstructure of the material studied after the heat treatment in a plane parallel to the rolling direction (vertical). The austenite phase is presented in Figure 4a, while the ferrite phase is depicted in Figure 4b. The colors represent the crystallographic orientation of the grains according to the color code inserts, which show the [001] standard triangle. The images were obtained by means of an automated electron backscatter diffraction (EBSD) analysis. In order to execute the EBSD analysis, a material sample was prepared by grinding and electrolytic etching. Mechanical properties of the heat-treated condition (HTC) and the as-received condition (ARC) of the duplex stainless steel were investigated by means of tensile tests in rolling direction. The mechanical properties are presented in Table 2.

**Figure 5:**
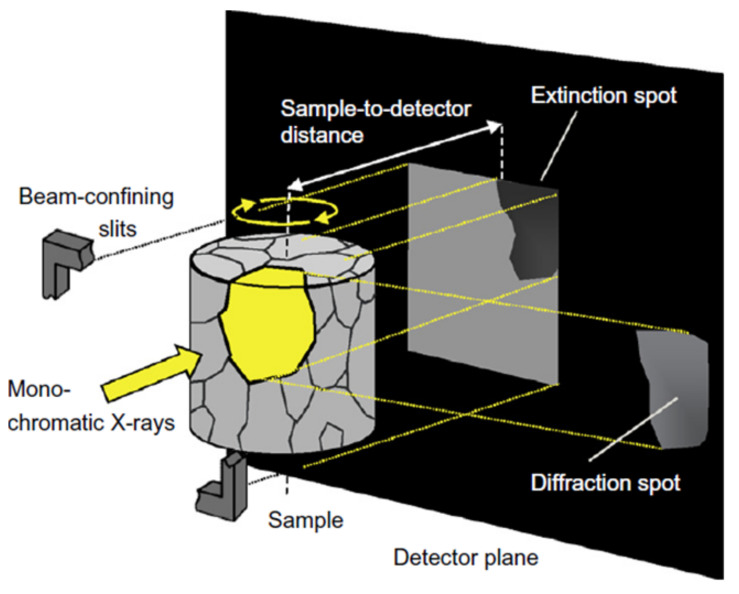
Principle of diffraction contrast tomography [13].

For the application of the diffraction contrast tomography (DCT) technique, a polycrystalline sample was mounted on a rotating sample holder and irradiated by means of a monochromatic X-ray beam (Figure 5). The energy of the X-ray beam had to be sufficiently high or the irradiated sample had to be sufficiently thin that enough X-ray radiation could transmit and that multiple diffraction was prohibited. Crystal planes of single grains diffracted the X-ray beam when fulfilling the Bragg condition. Hereby, single diffraction spots were generated on a semiconductor plate detector, which was positioned behind the sample in the direction of the incident beam. Because the diffracted X-ray beams did not reach the detector in the direction of the incident beam, an extinction spot was generated at the projected area of the diffracting grain in the direction of the incident beam at the detector. Furthermore, a diffraction spot was generated at the position where the diffracted beam reached the detector. The sample was rotated in small angle increments (0.1°) by, in total, 360° around the cylinder axis, and after each rotation step, a diffraction pattern was recorded by the detector. By means of the positions of the diffraction spots in the diffraction patterns and the corresponding rotation angles of the diffraction pattern images, the three-dimensional geometry and crystallographic orientation of each grain could be reconstructed. For this purpose, a sophisticated mathematical algorithm applied. Unfortunately, positioning all reconstructed grains at their correct position inside the sample volume could lead to a grain arrangement that does not represent the irradiated volume perfectly. Rather, occasionally, the reconstruction process gave rise to overlapping of several grains, or voids might be generated, affecting the accuracy of the determination of the local grain boundary orientation [12]. This drawback was partially mitigated by using the 3D phase reconstructions resulting from holotomography, a technique explained in detail in [7]. Due to different absorption coefficients of different phases (here: austenite and ferrite), contrast differences were generated on the semiconductor plate detector.

In principle, the setup for the phase contrast tomography (PCT) technique was very similar to that of DCT. The sample was rotated in small angle increments by, in total, 360° with respect to its axis, and at each angle, a contrast image was recorded. By overlapping the single contrast images according to the corresponding rotation angles, a three-dimensional volume reconstruction of the individual phases could be generated. In contrast to the microstructure reconstruction, which was generated by means of the DCT technique, as has been described before, the information of the crystallographic orientation of grains and the information about intra-phase grain boundaries were missing in the data, which was obtained by means of the PCT technique. However, the reconstructions that were generated by PCT were more precise regarding the positions of phase boundaries. For a more detailed description of the PCT technique the reader is referred to [14].

**Figure 6:**
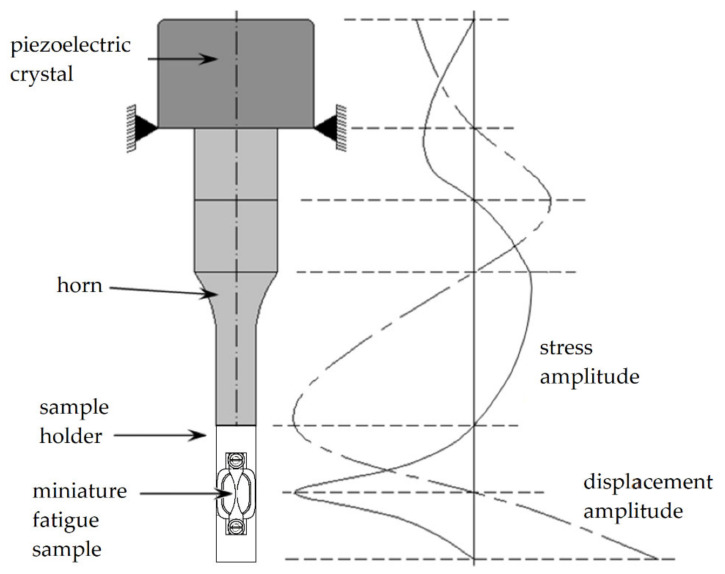
Ultrasonic fatigue testing equipment according to [16], in combination with a newly developed sample holder for miniature fatigue samples suitable for DCT and PCT.

The cyclic deformation experiments were executed by means of ultrasonic fatigue testing [15] at a testing frequency of about 20 kHz (Figure 6). The tests were carried out at room temperature and in laboratory atmosphere. A sinusoidal mechanical stress wave was generated by means of a piezoelectric crystal, amplified by means of a horn in its amplitude, and introduced into a fatigue sample holder, on which a miniature fatigue sample was mounted. Resonance occurred if the eigenfrequency of the assembly of miniature fatigue sample and sample holder was identical to the excitation frequency of the piezoelectric crystal. Then, the required stress amplitude in the area of minimum cross section of the miniature fatigue sample was generated. A control system kept this amplitude constant. Due to heat generation during the fatigue tests, a pulse-pause mode (100 ms/1200 ms) and an air-cooling system were required. Hereby, the temperature rise in the fatigue sample was restricted to maximum 5 °C. The chosen pulse-pause mode led to an effective testing frequency of about 1.5 kHz. Hereby, a testing of very high numbers of load cycles was possible in a reasonable testing time (e.g., one billion load cycles in about 7.5 days).

## 3. Some Numerical Details

The user-defined material subroutine UMAT, which was developed by Huang [17] for the commercial finite-element program ABAQUS, was applied in this study. This subroutine assumed that the plastic shear on single slip systems was determined by the elastic shear stress acting on the corresponding slip system. The plastic shear rates ${\dot{\gamma }}_{pl}$ on a single slip system were calculated by means of the following rate dependent flow law [18]:

(1) $${\dot{\gamma }}_{pl}=\dot{a}\;{\left(\frac{\tau }{\tau_{f}}\right)}^{m}$$

where $\dot{a}$ is the reference value of the plastic shear rate, which is reached when the shear stress *τ* equals the frictional shear stress *τ_f_*. By means of the power law exponent *m,* the course of the function ${\dot{\gamma }}_{pl}=f(\tau )$ is determined. An exponent value *m*→∞ corresponds to rate-independent material behavior [18]. The plastic shear was determined by means of numerical integration of the plastic shear rate over the time increment Δ*t* as follows:

(2) $$\Delta \gamma_{pl}=\Delta t\left(\left(1-\theta \right)\;{\dot{\gamma }}_{pl,t}+\theta \;{\dot{\gamma }}_{pl,t+\Delta t}\right)$$

The implicit integration parameter *θ* determines which fraction of the plastic shear rate at the end of a time increment is considered. The parameter can have a value between 0 and 1, whereas a value between 0.5 and 1 is recommended [19]. The plastic shear is iteratively calculated during a time increment. The iteration ends when the calculated plastic shear for a time increment is lower than a user-defined value Δ*γ_p_*_l,error_. In the framework of the simulations, which led to the results presented in the next section, the parameter values shown in Table 3 were used. For a more detailed description of the UMAT subroutine the reader is kindly referred to [17,20].

## 4. Results and Discussion

**Figure 7:**
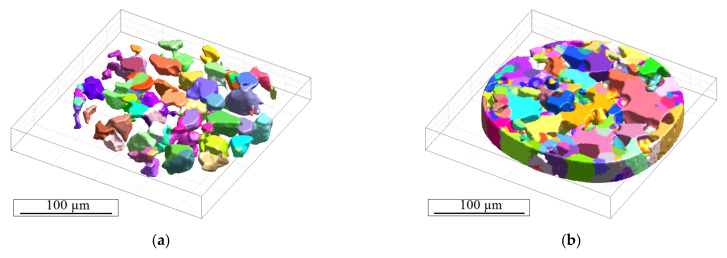
Reconstruction of (**a**) the austenite and (**b**) the ferrite grain microstructure.

In order to reconstruct the microstructure of the investigated duplex stainless steel, the advantages of both X-ray tomography techniques described in Section 2 were combined. The reconstructed 3D-microstructure of the austenite phase (Figure 7a) and of the ferrite phase (Figure 7b) were generated by combining the DCT volume data with the PCT volume data. Before doing this, the overlapping grain volume of the DCT reconstruction data was deleted.

**Figure 8:**
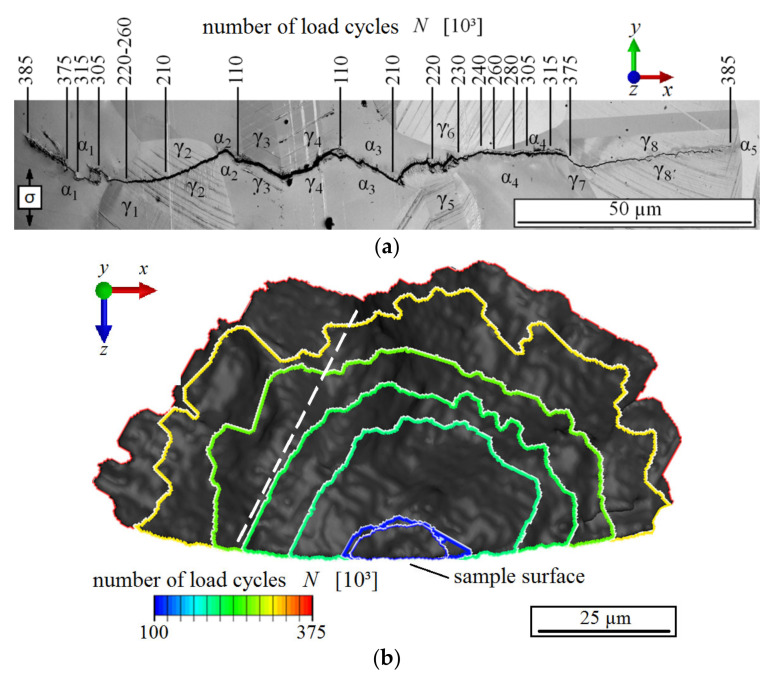

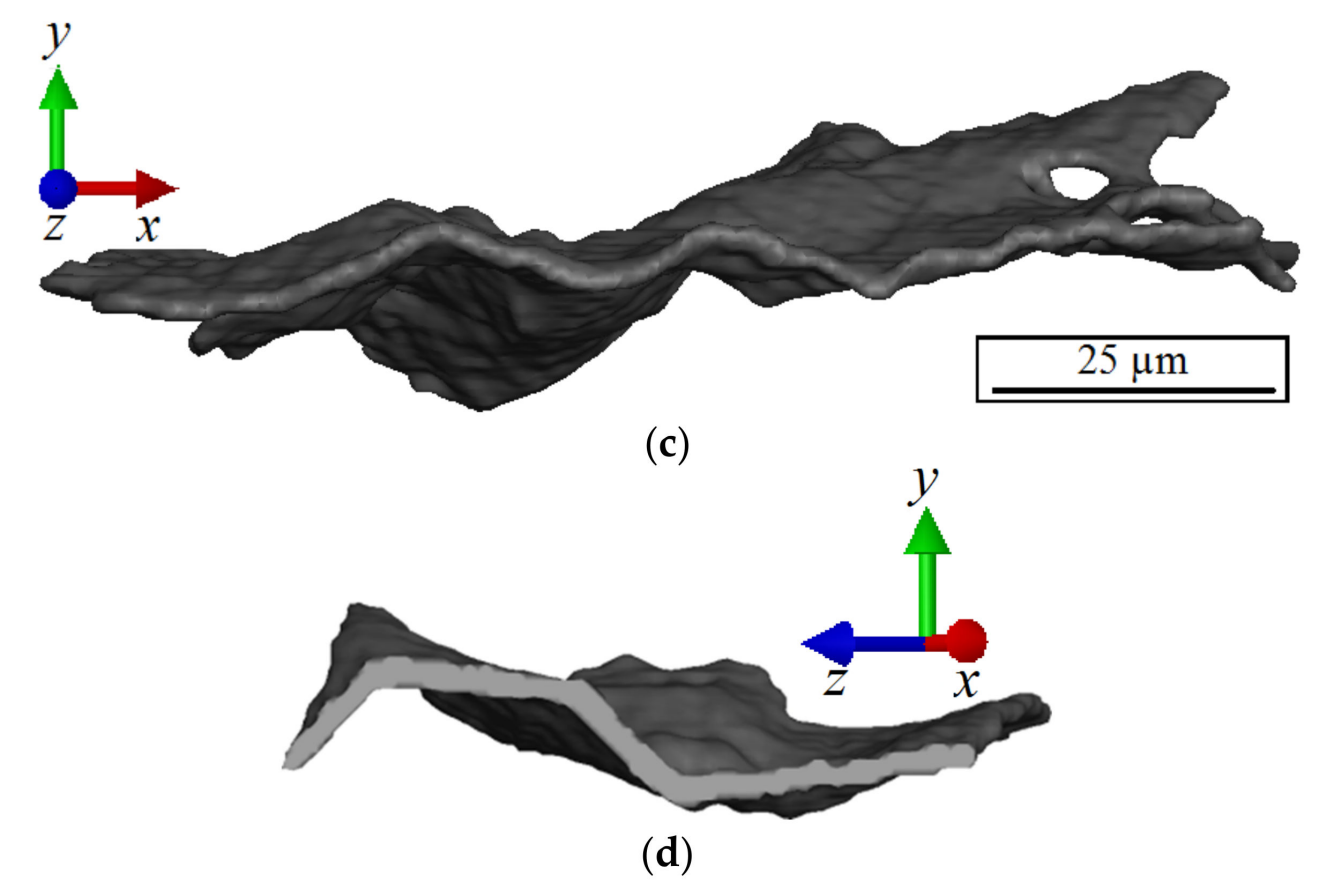
(**a**) Short fatigue crack propagation at sample surface. (**b**) Fracture surface of the fatigue crack shown in a) as a function of the number of load cycles (view in loading direction). (**c**) 3D geometry of the fatigue crack (view perpendicular to sample surface). (**d**) Profile of the fatigue crack along the dashed line in (**b**).

A fatigue crack at the surface of a miniature sample, to which ultrasonic fatigue testing at 20 kHz was applied, is shown in Figure 8a. The fatigue sample was fatigued at a stress amplitude of 400 MPa. The test was stepwise interrupted in order to monitor the short fatigue crack propagation by means of optical microscope images, which were taken after different loading cycles. The fatigue crack presented in Figure 8 initiated at two nucleation sites, i.e., (i) at the phase boundary between the austenite grain γ_3_ and the ferrite grain α_2_ and (ii) at the phase boundary between the austenite grain γ_4_ and the ferrite grain α_3_. The transcrystalline crack nuclei grew together and subsequently propagated in both directions as a consequence of further cyclic deformation. The crack growth was decelerated, when a crack tip approached a phase boundary, such as in the case of the phase boundary between the austenite grain γ_7_ and the ferrite grain α_4_. An acceleration of crack propagation was always detected when the crack overcame the boundary. At the phase boundary between the austenite grain γ_1_ and the ferrite grain α_1_, the crack was even temporarily stopped.

By means of the phase contrast tomography technique and the commercial 3D data visualization software AVIZO FIRE, the crack shown in Figure 8a was examined nondestructively and represented three-dimensionally after different numbers of load cycles. In order to execute the tomography investigations, the miniature sample had to be removed intermittently from the ultrasonic test rig and precisely placed and mounted into the synchrotron beam. Following each tomography experiment, the miniature sample was installed back again in the ultrasonic fatigue testing device in order to continue the cyclic loading, and so forth. The loading parameter values of the ultrasonic fatigue equipment were thoroughly kept constant during the whole experiment.

The crack front after a predefined numbers of loading cycles is displayed in the view direction parallel to the sample axis in Figure 8b. Clearly, the crack propagates roughly in a half-elliptical shape. However, the crack propagation rate obviously shows significant local differences. The crack is presented in Figure 8c after 3.75∙10^5^ loading cycles in the view direction perpendicular to the sample surface. Furthermore, Figure 8d shows the crack profile along the dashed white line delineated in Figure 8b. Both images (Figure 8c,d) clearly show the characteristic zigzag path that is typical of propagation of microstructurally short fatigue cracks, which propagate along single slip planes under predominant local single-slip conditions. The changes of the propagation direction at grain or phase boundaries are caused by changes in the crystallographic orientation (not shown here).

**Figure 9:**
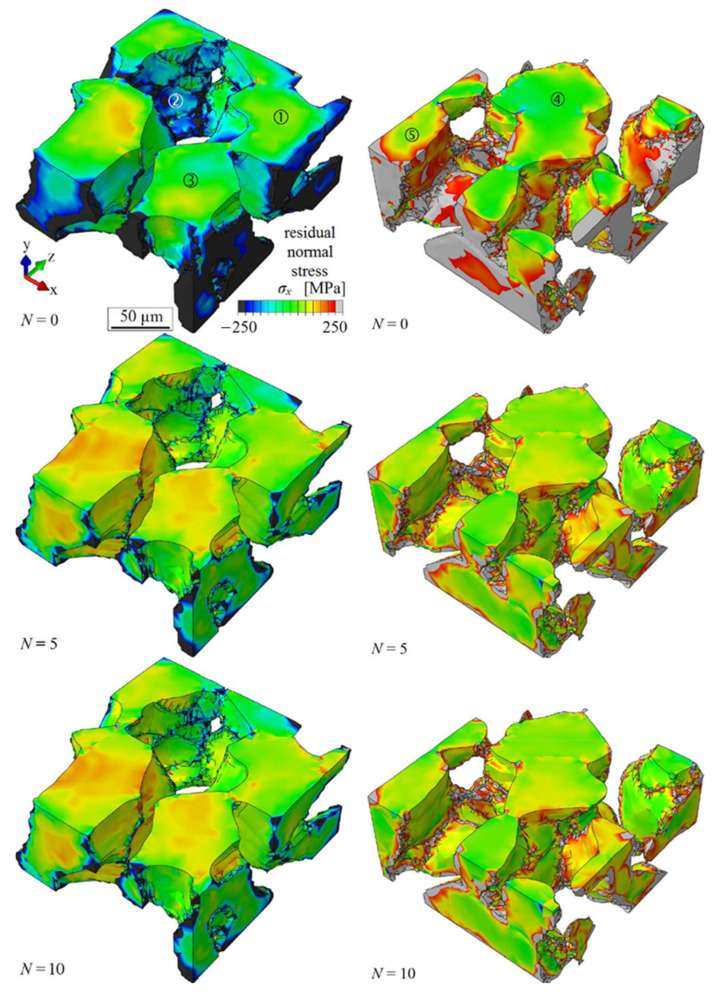
Development of residual stress in the ferrite phase (**left**) and the austenite phase (**right**) after different simulated numbers of load cycles.

A three-dimensional microstructure of the investigated duplex stainless steel is presented in Figure 9. The ferrite phase is shown at the left side and the corresponding austenite phase is depicted at the right side. The geometry of the grains and the phase distribution was determined by means of the combination of DCT and PCT (see Section 2). The geometry information was discretized by tetrahedral finite elements by means of the commercial image processing software AVIZO FIRE. The experimentally determined phase affiliation of each grain and the individual crystallographic grain orientation were considered. Moreover, anisotropic elasticity and lattice-type-specific crystal plasticity were taken into account in the stress distribution calculations (Section 3). The volume fraction of both phases was confirmed to be about 50% each. In order to consider the behavior of the surrounding of the depicted volume of the microstructure in a reasonable approach, this section was embedded in a frame consisting of finite elements with isotropic-elastic material behavior (Young’s modulus *E* = 197 MPa and Poisson’s ratio *ν* = 0.3). This surrounding frame is not shown in Figure 9 for the sake of clarity.

The nodes at the left face of the model were fixed in x-direction. One node at the upper left corner of the model was fixed in z-direction and one node at the lower left corner was additionally fixed in z- and y-direction to avoid rigid body motions. To define the starting condition, the residual stress distribution resulting from the cooling at the end of the initial annealing process was calculated. For this purpose, the quenching process from 1050 °C to room temperature was numerically simulated applying temperature-dependent elastic constants *E*_11_, *E*_12_ and *E*_44_; a temperature-dependent thermal expansion coefficient α; temperature-dependent microstructural frictional shear stresses of both phases; and the temperature-dependent Young’s modulus *E* for the isotropic-elastic frame (details are provided in [2]). Furthermore, the first order residual stresses, which were experimentally determined by conventional X-ray diffraction measurements, were considered in the microstructure section of Figure 9 by applying a load of 50.2 MPa in tension at the nodes at the right face of the model and 21 MPa in compression at the upper and lower face of the model. The simulation of the quenching process was followed by a simulation of the effect of load cycles. For this, a cyclic normal stress amplitude of 350 MPa was applied at the nodes at the right face of the model.

Because of the higher coefficient of thermal expansion of the austenite phase as compared to the ferrite phase, the starting condition after quenching was characterized by dominating compressive internal stresses in the ferrite and tensile stresses in the austenite. The distribution of the simulated residual normal stress in load direction *σ*_x_ in the ferrite phase after different numbers of load cycles *N* is presented at the left side of Figure 9. Some grains showed no change of the initial residual stresses with increasing numbers of load cycles (e.g., position 1). However, initial compressive residual stresses in some other grains were significantly reduced during the first loading cycles, but less during further cycles (e.g., position 2). Furthermore, significant tensional residual stresses built up during the first loading cycles in some grains, which reached a saturation state during the following load cycles—for example position 3.

At the right side of Figure 9, the simulated residual normal stress in load direction *σ*_x_ is presented for the austenite phase after different numbers of load cycles *N*. Some grains showed no significant change of residual stress with increasing numbers of loading cycles (e.g., position 4). However, a decrease of initial tensile residual stress was observed in the other grains (e.g., position 5).

Basically, the expected equalization of the residual stresses by means of cyclic plastic loading could be confirmed. However, in some ferritic grains, a localized stress increase took place that seemed to promote crack initiation. It should be emphasized that this type of calculations were found to explain the location of crack initiation [3]. In this respect, 3D-calculations on the basis of an extruded microstructure, which do not take the third-dimension correctly into account, are much less unerring.

## 5. Conclusions

Advanced diffraction contrast tomography (DCT) and phase contrast tomography (PCT) were combined and successfully applied to an austenitic–ferritic duplex stainless steel. It was shown that a three-dimensional microstructure of a metallic material that contained two phases with different crystal structures could be reconstructed nondestructively. Besides the information on the phase distribution and grain microstructure of the material, this sophisticated technique additionally provided crystallographic orientation data of the grains.

The obtained data were used to create realistic three-dimensional finite-element models of the microstructure. By means of a crystal plasticity finite-element analysis, experimentally determined first and second order residual stresses caused by the manufacturing process of the material were simulated. The results of subsequent cyclic deformation simulations were found to be in good agreement with experimental results on the evolution of initial residual stress on the microstructural level and strongly support the identification of the critical fatigue crack nucleation sites. In the duplex stainless steel investigated, solely in ferrite grains, residual stresses built up locally due to cyclic deformation promoting crack nucleation in this phase in accordance to the experimental observations.

By applying a suitable fatigue damage parameter (e.g., the one proposed in ref. [2]), crystal plasticity finite-element simulations based on three-dimensional microstructures, which were obtained nondestructively by means of DCT and PCT, provide a powerful instrument to analyze and quantitatively judge separately the effect of microstructural parameters regarding their influence on the fatigue behavior of a material. Furthermore, cyclic loading experiments intermitted by DCT and PCT analyses enable a 3D visualization and re-modeling of crack propagation behavior, being, in particular, useful for an improved understanding of the mechanisms which control the propagation of microstructural short fatigue cracks. The results of the present work clearly demonstrate the significance of phase and grain boundaries as microstructural barriers for short fatigue crack growth, both at the surface and in the interior of multi-phase alloys.

## Figures and Tables

**Table 1:** Chemical composition of the investigated duplex stainless steel (mass concentration in %) [11].

C	Cr	Ni	Mo	N	Fe
0.03	21.0–23.0	4.5–6.5	2.5–3.5	0.1–0.22	rest

**Table 2:** Mechanical properties of the heat-treated condition (HTC) and the as-received condition (ARC) of the investigated duplex stainless steel in rolling direction [11].

Condition	Young’s Modulus [GPa]	0.2% Yield Strength [MPa]	Tensile Strength [MPa]	Elongation at Fracture [%]
HTC	197	535	770	59
ARC	197	720	870	33

**Table 3:** Parameters used in this work for the UMAT subroutine [11,17].

Parameter	Symbol	Value	Dimension
reference shear rate	$\dot{a}$	10^−3^ [17]	1/s
power law exponent	*m*	20 [17]	–
frictional shear stress of austenite phase	*τ* *_fγ_*	68 [11]	MPa
frictional shear stress of ferrite phase	*τ* *_fα_*	99 [11]	MPa
implicit integration parameter	*Θ*	0.5 [17]	–
iteration stop criterion	Δ*γ_pl,error_*	10^−5^ [17]	–

## Data Availability

The data presented in this study are available on request from the corresponding author.
